# Long-term PM2.5 exposure and incident heart failure in the UK Biobank: relative and absolute risk estimates

**DOI:** 10.3389/fpubh.2026.1864868

**Published:** 2026-09-14

**Authors:** Lei Wang, Ling Ma, Huan Liu, Qian Zhu

**Affiliations:** 1 Department of Vascular Surgery, General Hospital of Ningxia Medical University, Yinchuan, China; 2 Departments of Medicine, University of Rochester School of Medicine and Dentistry, Rochester, NY, United States; 3 Medical Laboratory, Hospital of Cardiovascular and Cerebrovascular Diseases, General Hospital of Ningxia Medical University, Yinchuan, China

**Keywords:** air pollution, cohort study, environmental exposure, heart failure, PM2.5, UK Biobank

## Abstract

**Background:**

To investigate the association between long-term air pollution exposure and incident heart failure in the UK Biobank and further explore potential effect modification by cardiometabolic vulnerability.

**Methods:**

We conducted a prospective cohort study among 149,410 UK Biobank participants free of heart failure and ischemic stroke at baseline. Annual mean concentrations of fine particulate matter (PM2.5), nitrogen dioxide (NO2), and particulate matter ≤10 μm (PM10) were assigned using land-use regression models. Incident heart failure and ischemic stroke were ascertained through linkage to hospital and death records. Cox proportional hazards models were used to estimate hazard ratios (HRs) per interquartile range (IQR) increase in pollutants with sequential adjustment for demographic, socioeconomic, lifestyle, and cardiometabolic factors. Effect modification by diabetes, chronic kidney disease, obesity, multimorbidity, and C-reactive protein was assessed using interaction terms.

**Results:**

During a median follow-up of 17.6 years (interquartile range 14.4–20.3), 4,964 incident heart failure events and 3,795 ischemic stroke events occurred. PM2.5 was associated with a higher risk of incident heart failure after full adjustment (HR per IQR 1.045, 95% CI 1.005–1.086; *p* = 0.027). Participants in the highest quartile of PM2.5 exposure also had a higher risk of heart failure than those in the lowest quartile (HR 1.133, 95% CI 1.037–1.238; P-trend = 0.005). Dose–response analyses suggested an approximately linear relationship between PM2.5 and heart failure risk, with no strong evidence of non-linearity. No statistically significant interaction was observed between PM2.5 and cardiometabolic or inflammatory vulnerability factors (all *P* for interaction >0.10). In contrast, no significant associations were observed between air pollution metrics and ischemic stroke. Standardized absolute risks of heart failure were low, with a 20-year absolute risk difference of approximately 0.2 per 100 persons between the highest and lowest PM2.5 exposure groups.

**Conclusion:**

In this large population-based cohort, long-term exposure to PM2.5 was associated with a modestly increased risk of incident heart failure. We found no strong evidence that this association differed across major cardiometabolic or inflammatory vulnerability groups. Although the absolute risk difference was small at the individual level, these findings add to the evidence linking long-term PM2.5 exposure to incident heart failure.

## 1 Introduction

Cardiovascular diseases remain a leading cause of morbidity and mortality worldwide, accounting for a substantial and growing proportion of global disease burden (1, 2). Among these, heart failure represents a particularly important clinical and public health challenge. It is a chronic, progressive syndrome associated with high rates of hospitalization, impaired quality of life, and considerable healthcare costs, especially in ageing populations (3). Despite advances in pharmacological and device-based therapies, the incidence of heart failure continues to rise, and traditional risk factors such as hypertension, diabetes, obesity, and coronary artery disease do not fully explain its population-level occurrence (4, 5). Identifying additional modifiable risk factors for heart failure, therefore remains a major public health priority. Furthermore, ischemic stroke is another major contributor to cardiovascular morbidity and long-term disability (6). Although well-established risk factors such as atrial fibrillation, hypertension, and diabetes play a central role in stroke pathogenesis (7), a substantial proportion of stroke risk remains unexplained, and marked geographic and population-level heterogeneity persists. Thus, environmental and contextual exposures may contribute meaningfully to the risk of cerebrovascular disease.

Ambient air pollution has emerged as a pervasive environmental risk factor with potential relevance for cardiovascular health (8). Fine particulate matter (PM2.5), a major component of air pollution, has been consistently associated with increased cardiovascular morbidity and mortality in large-scale epidemiological studies (9), including elevated risks of ischemic heart disease, stroke, and cardiovascular death, even at concentrations below current regulatory standards (10). Mechanistic studies suggest that inhaled fine particles can penetrate deep into the alveoli and trigger systemic inflammation, oxidative stress, endothelial dysfunction, and autonomic imbalance, thereby promoting atherosclerosis and other pathophysiological processes relevant to cardiovascular disease (11–13).

While the association between PM2.5 and overall cardiovascular risk is well established (10), the magnitude and clinical relevance of this association (14–16), particularly in terms of absolute risk, remain less clearly defined. Most prior studies have focused on short-term exposures and acute heart failure hospitalizations, whereas fewer large-scale population-based studies have examined whether chronic exposure contributes to the onset of new heart failure in the general population. Similarly, although many cohort studies and meta-analyses have reported positive associations between PM2.5 and stroke risk (10), findings have been heterogeneous across regions and populations, and residual confounding remains a concern. Cardiometabolic conditions such as diabetes, chronic kidney disease, and obesity are well-established risk factors for both heart failure and stroke and may plausibly modify the cardiovascular effects of air pollution through shared biological pathways involving inflammation, oxidative stress, and metabolic dysregulation (17). However, relatively few large population-based studies have systematically investigated whether these vulnerability profiles amplify the cardiovascular risks associated with long-term air pollution exposure.

In this study, we used data from the UK Biobank to examine the association between long-term PM2.5 exposure and incident heart failure in a large prospective population-based cohort. We further assessed the dose–response relationship and explored whether this association differed according to cardiometabolic and inflammatory vulnerability profiles, including diabetes, chronic kidney disease, obesity, multimorbidity, and C-reactive protein levels. In addition to relative risk estimates, we also evaluated standardized absolute risks over extended follow-up to better characterize the potential public health relevance of the observed association. Ischemic stroke and other air pollution metrics were evaluated as secondary analyses.

## 2 Methods

### 2.1 Study design and population

We conducted a prospective population-based cohort study using data from the UK Biobank. The UK Biobank is a large ongoing cohort that enrolled over 500,000 participants aged 40–69 years between 2006 and 2010 across the United Kingdom. At baseline, participants completed detailed questionnaires, physical measurements, and provided biological samples. Longitudinal follow-up for health outcomes is available through linkage to national hospital admission, disease registry, and death records. Of the more than 500,000 UK Biobank participants, 210,239 had the core data required to construct the air-pollution cardiovascular cohort, including residential air-pollution measures, baseline assessment date, outcome follow-up information, and the linked clinical datasets used in this analysis. Participants without these core data were not eligible for cohort construction.

In this study, we included participants with available data on air pollution exposure, baseline assessment date, follow-up or censoring date, and relevant covariates. We excluded individuals with a history of heart failure or ischemic stroke at baseline. We further excluded participants with missing or implausible follow-up times and those with missing values for covariates required for the fully adjusted models. The final analytical cohort consisted of 149,410 participants. The UK Biobank study was approved by the North West Multi-centre Research Ethics Committee, and all participants provided written informed consent. The present analysis was conducted under an approved UK Biobank application.

### 2.2 Exposure assessment

Annual average concentrations of particulate matter with aerodynamic diameter ≤2.5 μm (PM2.5), nitrogen dioxide (NO2), and particulate matter ≤10 μm (PM10) were estimated using land-use regression models developed as part of the European Study of Cohorts for Air Pollution Effects (ESCAPE) and related projects. These estimates were assigned to participants according to their residential address, and the 2010 annual average concentration was used as the primary exposure metric in this study. We treated these values as a proxy for near-baseline long-term ambient air pollution exposure rather than as time-varying exposure measures over follow-up. Air pollution exposures were modelled as continuous variables scaled per interquartile range (IQR) increase for regression analyses. In addition, we constructed a multipollutant score by standardizing PM2.5, NO2, and PM10 (when available) and calculating their mean. For descriptive analyses, PM2.5 was also categorized into quartiles.

### 2.3 Outcome ascertainment

The primary outcome was incident heart failure, identified through linkage to hospital inpatient records and disease registries using International Classification of Diseases, 10th Revision (ICD-10) code I50. The secondary outcome was incident ischemic stroke, identified using ICD-10 code I63. Follow-up time was calculated from the date of the baseline assessment to the date of first diagnosis of the outcome of interest, date of death, or end of administrative follow-up (31 December 2024), whichever occurred first.

### 2.4 Covariates

We included *a priori* selected covariates based on existing literature and biological plausibility, including age at recruitment, sex, socioeconomic status (Townsend deprivation index), smoking status, alcohol intake frequency, physical activity level, body mass index (BMI), obesity (BMI ≥ 30 kg/m^2^), diabetes, chronic kidney disease (CKD), and C-reactive protein (CRP). Diabetes was defined based on self-report and hospital records prior to baseline. CKD was defined as an estimated glomerular filtration rate (eGFR) < 60 mL/min/1.73 m^2^, calculated using serum creatinine and the CKD-EPI equation. CRP was dichotomized as high or low based on the cohort median. We also adjusted for traffic-related exposure indicators, including proximity to major roads and traffic intensity where available. For subgroup analyses, we further defined a simplified cardiometabolic multimorbidity indicator as the presence of at least two of the following baseline conditions: obesity, diabetes, and CKD. This composite variable was used to reflect cardiometabolic vulnerability in the context of air pollution susceptibility, rather than conventional multimorbidity across all chronic conditions.

### 2.5 Statistical analysis

We summarized baseline characteristics by quartiles of PM2.5 using means, medians, and proportions as appropriate. We used Cox proportional hazards regression models to estimate hazard ratios (HRs) and 95% confidence intervals (CIs) for the association between air pollution and incident heart failure and ischemic stroke. We fitted sequential models with increasing levels of adjustment: Model 1: adjusted for age and sex; Model 2: additionally adjusted for socioeconomic status, smoking, alcohol intake, and physical activity; Model 3 (primary model): further adjusted for BMI, obesity, diabetes, CKD, CRP, and traffic-related variables. Air pollution exposures were modeled per IQR increase and, in secondary analyses, as quartiles. Analyses were conducted using complete cases for the covariates included in each model; therefore, the analytical sample size decreased with increasing covariate adjustment.

### 2.6 Dose–response and non-linearity

We assessed potential non-linear associations between PM2.5 and heart failure using restricted cubic splines with four degrees of freedom. We compared spline models with linear models using likelihood ratio tests to assess evidence for non-linearity.

### 2.7 Effect modification

We examined effect modification by cardiometabolic and inflammatory vulnerability factors, including diabetes, CKD, obesity, multimorbidity, and CRP level, by fitting interaction terms between PM2.5 and each modifier in separate Cox models. We reported stratum-specific HRs and *p* values for interaction.

### 2.8 Absolute risk estimation

We estimated standardized absolute risks of heart failure at 15 and 20 years using a g-formula approach based on fitted Cox models. We predicted risks under hypothetical scenarios in which all participants were assigned to the lowest or highest PM2.5 quartile, while keeping covariates at their observed values. Absolute risk differences (ARDs) were calculated as the difference in standardized risks between exposure groups. We used non-parametric bootstrap resampling (300 repetitions) to obtain 95% confidence intervals.

### 2.9 Sensitivity analyses

We conducted several sensitivity analyses to assess the robustness of the primary findings, including: (1) excluding events occurring within the first 2 years of follow-up to reduce potential reverse causation; (2) restricting analyses to never smokers; (3) fitting two-pollutant models including both PM2.5 and NO2; and (4) restricting analyses to participants without diabetes or without chronic kidney disease. Detailed definitions of all study variables and data sources are shown in Supplementary Table S1.

### 2.10 Software

All analyses were conducted using R (version 4.2.3; R Foundation for Statistical Computing, Vienna, Austria). Statistical significance was defined as a two-sided *p-*value <0.05.

## 3 Results

### 3.1 Study population and follow-up

We merged air pollution, clinical, biomarker, and outcome data for 210,239 participants with complete information on baseline date, censoring date, and air pollution exposure. After application of the outcome-related eligibility criteria, 206,829 participants comprised the outcome-free cohort used for the minimally adjusted analyses. Of these, 149,410 had complete information for all covariates required for the fully adjusted model and constituted the primary fully adjusted analytical cohort (Figure 1). During a median follow-up of 17.6 years (interquartile range 14.4–20.3 years), we observed 4,964 incident heart failure events and 3,795 incident ischemic stroke events. The crude incidence rate of heart failure in the analytical cohort was 2.12 per 1,000 person-years.

**Figure 1:**
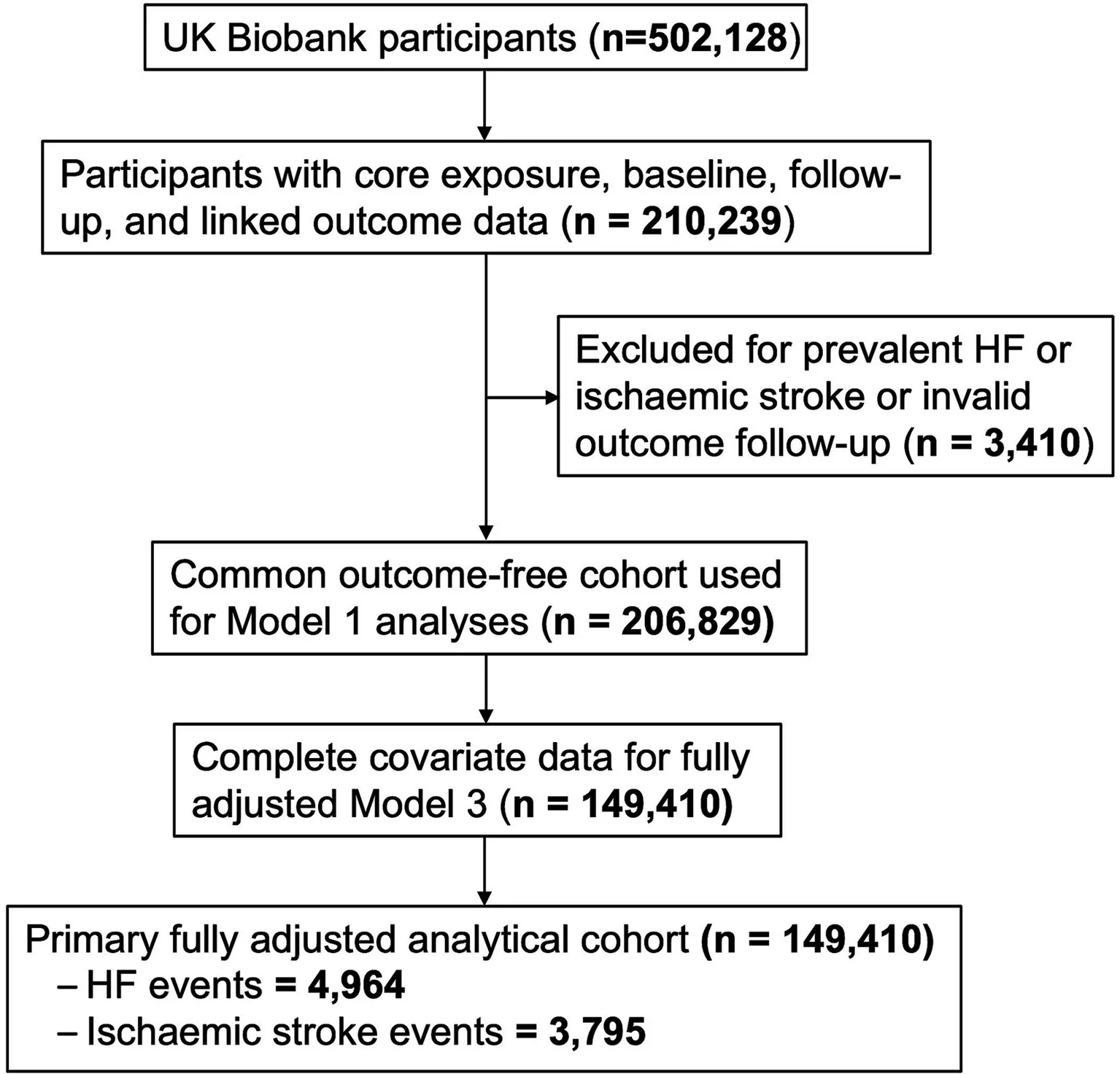
Flowchart of participant selection. Flowchart showing derivation of the common analytical cohort for heart failure and ischemic stroke analyses. From the UK Biobank source population, participants with the core exposure, baseline, follow-up, and linked outcome data required for cohort construction were identified. After outcome-related exclusions, 206,829 participants comprised the common outcome-free cohort used for minimally adjusted analyses. Of these, 149,410 participants with complete covariate information comprised the primary fully adjusted analytical cohort. Participants not included in the initial 210,239-person cohort lacked one or more core data elements required for cohort construction; exclusion categories at this stage were overlapping and therefore were not presented as mutually exclusive counts.

### 3.2 Baseline characteristics by PM2.5 exposure

Baseline characteristics of the 206,829 participants in the outcome-free cohort are shown across quartiles of annual mean PM2.5 concentration in Table 1. The fully adjusted analyses were subsequently restricted to 149,410 participants with complete covariate information. Participants in higher PM2.5 quartiles tended to have a less favorable cardiometabolic and socioeconomic profile. Specifically, they had slightly higher BMI, a higher prevalence of obesity and diabetes, higher CRP concentrations, and substantially greater socioeconomic deprivation as reflected by the Townsend index. Crude incidence rates of heart failure also increased across PM2.5 quartiles, from 1.821 per 1,000 person-years in the lowest quartile to 2.371 per 1,000 person-years in the highest quartile. These patterns suggested that higher PM2.5 exposure was associated with a more adverse baseline risk profile, indicating the potential for substantial confounding in unadjusted analyses.

**Table 1:** Baseline characteristics of the outcome-free cohort by quartiles of PM_2.5_ exposure (*n* = 206,829).

Variable	Q1	Q2	Q3	Q4
*n*	52,251	51,661	51,286	51,631
Age, years (mean)	57.011	56.680	56.170	55.421
Male, %	45.835	45.448	45.872	46.114
BMI, kg/m^2^ (mean)	27.042	27.280	27.491	27.489
Obesity, %	21.161	23.135	24.939	24.985
Diabetes, %	1.604	1.771	2.077	2.136
CKD, %	1.244	1.247	1.406	1.311
CRP, mg/L (median)	1.240	1.290	1.320	1.350
Townsend index (mean)	−2.787	−2.093	−1.277	0.569
Heart failure events, n	1,638	1868	1929	2,106
Heart failure incidence (per 1,000 PY)	1.821	2.098	2.196	2.371
Ischemic stroke events, *n*	1,335	1,336	1,424	1,501
Ischemic stroke incidence (per 1,000 PY)	1.484	1.500	1.620	1.688

Baseline characteristics are presented for the 206,829 participants in the outcome-free cohort used for the minimally adjusted analyses. Values are means, medians, or percentages unless otherwise indicated. The fully adjusted Model 3 analysis was restricted to 149,410 participants with complete covariate information. PY = person-years; CKD = chronic kidney disease; CRP = C-reactive protein.

### 3.3 Associations between air pollution and cardiovascular outcomes

In fully adjusted Cox proportional hazards models (Model 3), we found that PM2.5 was positively associated with incident heart failure (hazard ratio [HR] per interquartile range [IQR] increase 1.045, 95% CI 1.005–1.086; *p* = 0.027; Table 2). When PM2.5 was analyzed in quartiles, participants in the highest exposure quartile had a higher risk of heart failure compared with those in the lowest quartile (HR for Q4 vs. Q1 1.133, 95% CI 1.037–1.238; P-trend = 0.005), whereas no such gradient was observed for ischemic stroke (Supplementary Table S2). In contrast, we did not observe a significant association between NO2 and heart failure (HR 0.998, 95% CI 0.956–1.042; *p* = 0.937), nor for the multipollutant score (HR 1.021, 95% CI 0.986–1.058; *p* = 0.242). For ischemic stroke, none of the air pollution metrics showed statistically significant associations in the fully adjusted models, including PM2.5 (HR per IQR 1.027, 95% CI 0.982–1.073; *p* = 0.242), NO2, or the multipollutant score (Table 2).

**Table 2:** Associations of air pollution with incident heart failure and ischemic stroke.

Outcome	Exposure	HR	95% CI	*p* value
Heart failure	PM2.5 (per IQR)	1.045	1.005–1.086	0.027
Heart failure	NO2 (per IQR)	0.998	0.956–1.042	0.937
Heart failure	Multi-pollutant score (per 1 SD)	1.021	0.986–1.058	0.242
Ischemic stroke	PM2.5 (per IQR)	1.027	0.982–1.073	0.242
Ischemic stroke	NO2 (per IQR)	0.994	0.947–1.043	0.797
Ischemic stroke	Multi-pollutant score (per 1 SD)	1.002	0.964–1.042	0.911

Hazard ratios (HRs) were estimated using Cox proportional hazards models adjusted for age, sex, socioeconomic status, smoking, alcohol intake, physical activity, BMI, diabetes, CKD, and other covariates as specified in the Methods. IQR = interquartile range.

### 3.4 Sequential adjustment of the association

Sequential adjustment materially altered the estimated association between PM2.5 and incident heart failure (Supplementary Table S3). In the age- and sex-adjusted model, PM2.5 was associated with heart failure risk (HR 1.183, 95% CI 1.153–1.213; *p* < 0.001). This association was substantially attenuated after additional adjustment for socioeconomic and lifestyle factors (HR 1.022, 95% CI 0.986–1.058; *p* = 0.234), and then became modestly stronger and statistically significant after further adjustment for cardiometabolic factors and traffic-related variables (HR 1.045, 95% CI 1.005–1.086; *p* = 0.027). The marked attenuation from Model 1 to Model 2 indicates that socioeconomic and lifestyle factors accounted for a substantial portion of the age- and sex-adjusted association.

### 3.5 Dose–response relationship

Restricted cubic spline analysis showed no strong statistical evidence of non-linearity in the association between PM2.5 and incident heart failure (*P* for non-linearity >0.05; Figure 2). The fitted curve suggested a generally increasing association across the central portion of the observed exposure range, although confidence intervals widened at the exposure extremes. The observed PM2.5 range overlapped the 10 μg/m^3^ annual mean concentration target for England.

**Figure 2:**
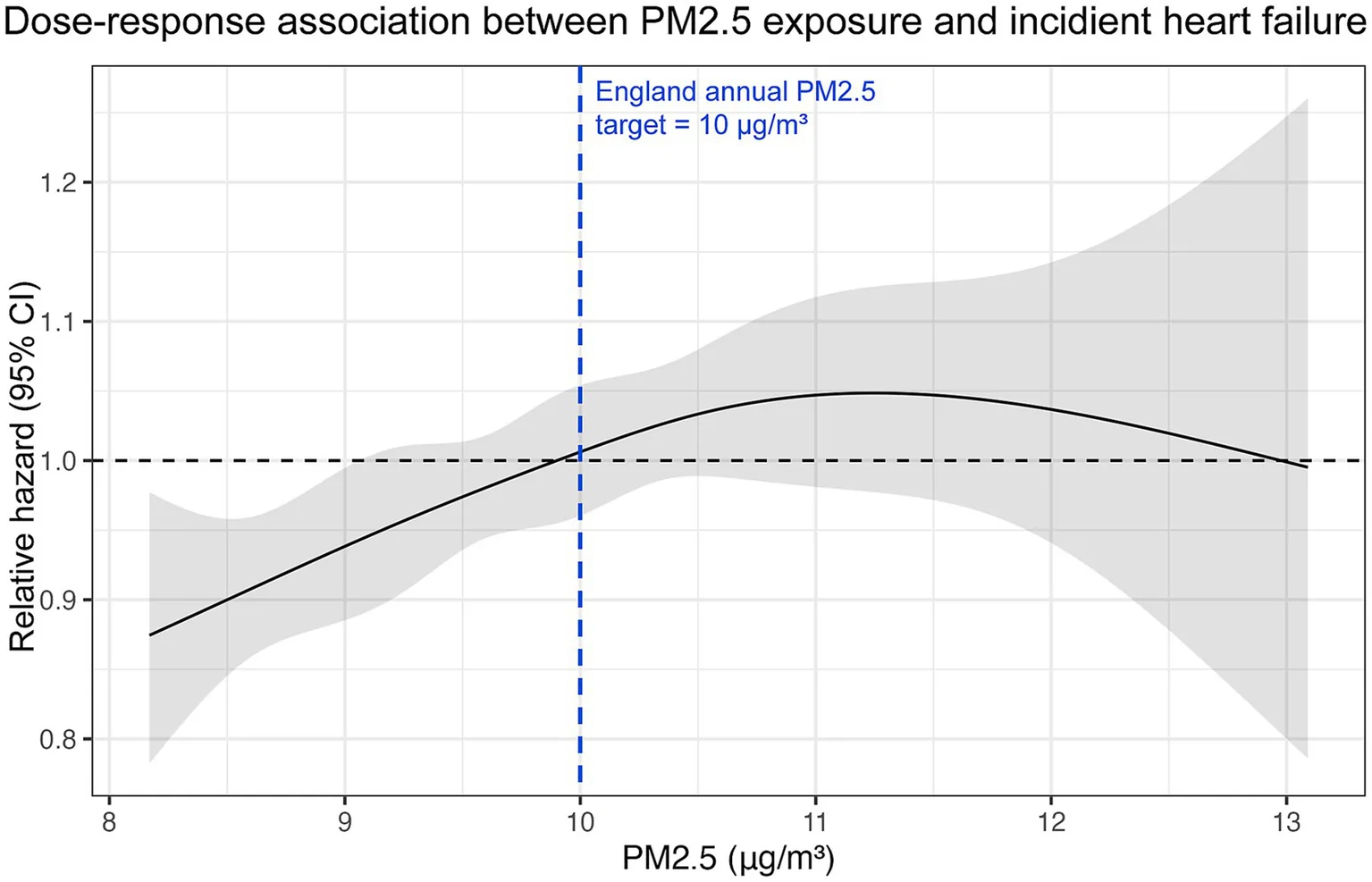
Dose–response association between PM2.5 exposure and incident heart failure. Restricted cubic spline showing the adjusted hazard ratio for incident heart failure according to annual mean PM2.5 concentration, with the median exposure used as the reference. The solid curve represents the hazard ratio and the shaded area represents the 95% confidence interval. The horizontal dashed line indicates an HR of 1.0. The vertical reference line at 10 μg/m^3^ indicates the annual mean PM2.5 concentration target for England. There was no strong statistical evidence of non-linearity (*P* for non-linearity >0.05).

### 3.6 Effect modification by cardiometabolic vulnerability

We further examined whether the association between PM2.5 and incident heart failure differed according to cardiometabolic and inflammatory vulnerability factors, including diabetes, CKD, obesity, multimorbidity, and CRP level (Figure 3 and Supplementary Table S4). Although point estimates appeared slightly higher in some lower-risk strata, formal tests for interaction did not provide statistical evidence of effect modification for any of the factors examined, including diabetes (*P* for interaction = 0.650), CKD (*p* = 0.444), obesity (*p* = 0.522), multimorbidity (*p* = 0.144), and CRP (*p* = 0.855). These findings provided no strong statistical evidence of effect modification across the examined subgroups.

**Figure 3:**
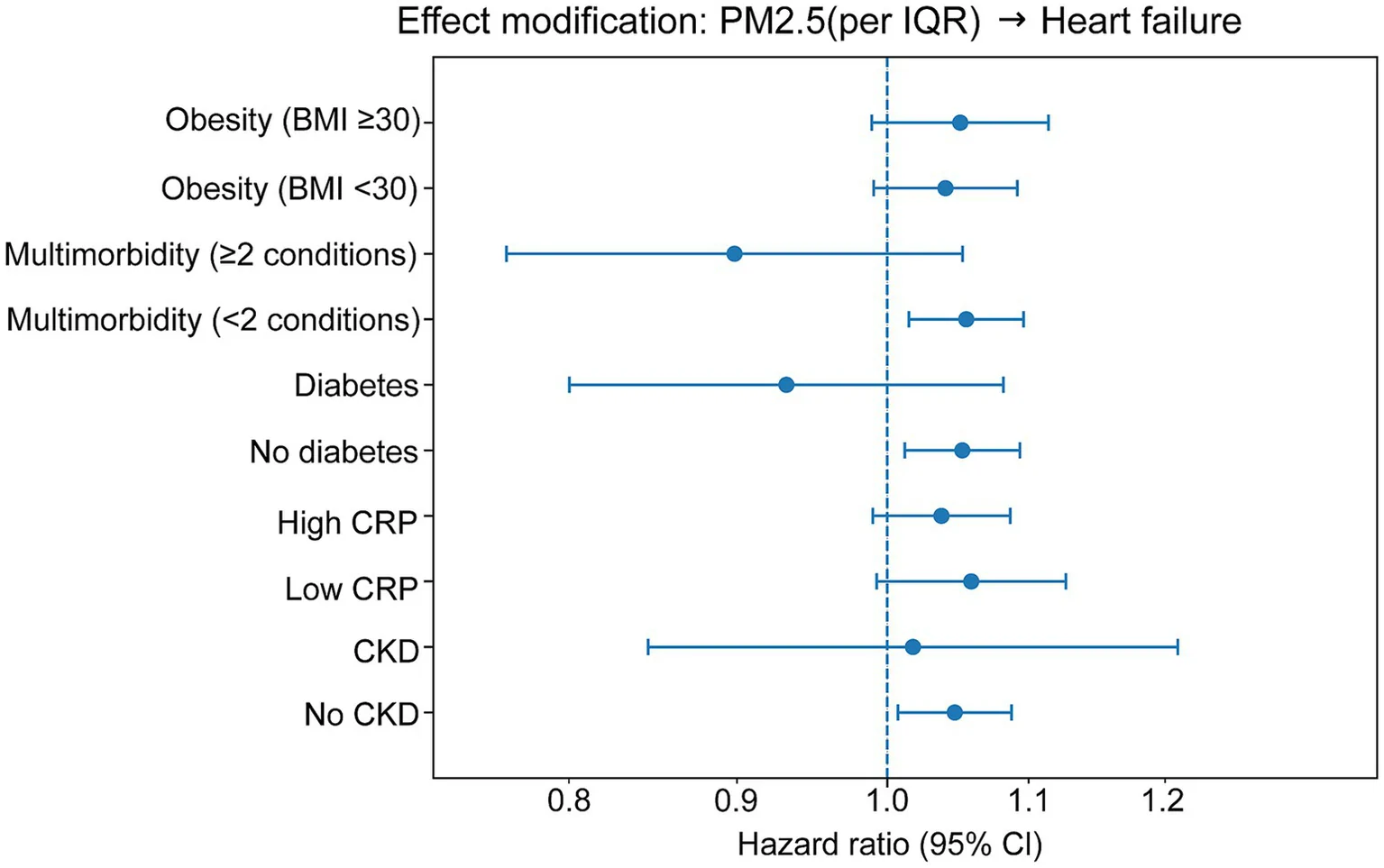
Association between PM2.5 and incident heart failure across vulnerability subgroups. Forest plot showing hazard ratios for incident heart failure per interquartile range increase in PM2.5 across subgroups defined by diabetes status, chronic kidney disease, obesity, multimorbidity, and C-reactive protein level. Points represent hazard ratio estimates and horizontal lines represent 95% confidence intervals. *p*-values for interaction between PM2.5 and each subgroup are shown. Models were adjusted for the same covariates as the primary model, except for the stratifying variable where appropriate.

### 3.7 Absolute risk differences

Standardized absolute risks of heart failure at 15 and 20 years by PM2.5 quartiles are shown in Supplementary Table S5. In the overall cohort, the standardized 15-year heart failure risk increased from 0.9 per 100 persons in Q1 to 1.1 per 100 persons in Q4, corresponding to an absolute risk difference (ARD) of 0.1 per 100 persons (95% CI 0.0–0.2). At 20 years, the corresponding ARD was 0.2 per 100 persons (95% CI 0.0–0.4). Among participants with diabetes, absolute risk estimates were higher, but confidence intervals were wide and overlapped between exposure groups, and the ARDs were not statistically significant.

### 3.8 Sensitivity analyses

Sensitivity analyses are presented in Supplementary Table S6. Overall, the direction of the association between PM2.5 and incident heart failure remained broadly consistent across alternative analytic strategies. After excluding heart failure events occurring within the first 2 years of follow-up, the association was modestly attenuated and no longer statistically significant (HR 1.035, 95% CI 0.994–1.077; *p* = 0.092). Among never smokers, the point estimate remained similar to the primary analysis and was slightly larger in magnitude, although the confidence interval was wider and included the null (HR 1.054, 95% CI 0.992–1.121; *p* = 0.091). In contrast, the association became stronger in the two-pollutant model including NO2 (HR 1.142, 95% CI 1.068–1.221; *p* < 0.001) and remained statistically significant in analyses restricted to participants without diabetes (HR 1.054, 95% CI 1.012–1.097; *p* = 0.011) or without CKD (HR 1.048, 95% CI 1.007–1.091; *p* = 0.020). These findings suggest that the observed association was generally directionally consistent, although its magnitude was modest and not uniformly statistically significant across all sensitivity analyses.

## 4 Discussion

In this large prospective cohort from the UK Biobank, we found that higher long-term PM2.5 exposure was associated with a modestly increased risk of incident heart failure after adjustment for a wide range of socioeconomic, lifestyle, and cardiometabolic factors. In contrast, we did not observe clear evidence for associations with ischemic stroke, NO2, or the multipollutant score in fully adjusted models, nor did we observe strong evidence of effect modification across the examined vulnerability subgroups.

Our findings are broadly consistent with prior epidemiological studies reporting positive associations between long-term PM2.5 exposure and heart failure incidence or hospitalization (18, 19). Several cohort studies from North America and Europe have shown that chronic exposure to fine particulate matter is associated with increased risk of heart failure events (20, 21), although effect sizes have generally been small and heterogeneous across populations. The magnitude of the association observed in our study (approximately 4–5% higher risk per IQR increase in PM2.5) is comparable to that reported in previous large cohorts and meta-analyses, supporting the overall consistency of the association with prior literature (21). In contrast, we did not observe a statistically significant association between PM2.5 and ischemic stroke after full adjustment, despite higher crude incidence rates across PM2.5 quartiles. This attenuation after adjustment suggests that socioeconomic, behavioral, and cardiometabolic factors account for a substantial proportion of the apparent crude association. Previous studies have shown that the PM2.5-stroke association is more heterogeneous and may be more sensitive to confounding and regional context than the association with coronary or heart failure outcomes (22, 23). Several factors may contribute to the discrepancy between our null findings for ischemic stroke and positive associations reported in some previous studies. These include differences in population characteristics, ambient PM2.5 concentration ranges and chemical composition, exposure-assessment methods, outcome definitions and ascertainment, duration of follow-up, and covariate adjustment. UK Biobank participants are also healthier and more socioeconomically advantaged than the general UK population, and the relatively limited exposure contrast in the UK may reduce the ability to detect a small association with stroke. Furthermore, the marked attenuation after socioeconomic and lifestyle adjustment in our analysis suggests that residual or differential confounding may contribute to differences across studies. Therefore, our null stroke result should not be interpreted as evidence that long-term PM2.5 exposure is unrelated to cerebrovascular risk in other populations.

For regulatory context, the WHO 2021 air quality guideline recommends an annual mean PM2.5 concentration of 5 μg/m^3^, whereas England has established an annual mean concentration target of 10 μg/m^3^. The PM2.5 concentrations observed in our cohort were approximately 8–13 μg/m^3^ and therefore did not extend to the WHO guideline level but did span the 10 μg/m^3^ England target. Within this observed range, spline analyses provided no strong statistical evidence of a discrete threshold. These findings should not, however, be interpreted as identifying a safe or harmful regulatory concentration because the analysis was observational and confidence intervals were wide at the extremes of the exposure distribution. Experimental and clinical studies suggest that inhaled fine particles induce systemic inflammation, oxidative stress, endothelial dysfunction, and autonomic imbalance, all of which can contribute to myocardial injury, ventricular remodeling, and progression toward heart failure. Chronic exposure may exert its effects through cumulative subclinical injury rather than through abrupt plaque rupture or thrombosis (24). These mechanisms are biologically relevant to heart failure, although our observational findings cannot establish why the associations differed between heart failure and stroke in this cohort.

Although the primary association between PM2.5 and incident heart failure remained statistically significant in the fully adjusted model, some sensitivity analyses yielded borderline or non-significant results, underscoring the modest magnitude of the observed association and the need for cautious interpretation. Excluding heart failure events occurring within the first 2 years of follow-up modestly attenuated the association from an HR of 1.045 to 1.035, with the confidence interval crossing unity. This attenuation may indicate that early events, including previously undiagnosed or subclinical heart failure present near baseline, contributed modestly to the primary association. However, the effect estimate remained positive and the confidence intervals substantially overlapped, providing no strong evidence that reverse causation or early subclinical disease fully explains the finding. In contrast, restriction to never smokers did not materially attenuate the effect estimate; the HR was slightly larger than in the primary analysis (1.054 versus 1.045), although the confidence interval was wider and included unity. This pattern is more consistent with reduced statistical precision in the smaller never-smoker subgroup than with disappearance of the association. Because the never-smoker analysis was a restriction-based sensitivity analysis rather than a formal interaction test, it cannot establish whether the PM2.5–heart failure association differs according to smoking status. Residual confounding by smoking-related factors also cannot be completely excluded. Taken together, these sensitivity analyses suggest that the direction of association was generally preserved, but the modest effect size and reduced precision in restricted analyses warrant cautious interpretation.

We did not observe strong statistical evidence that the association between PM2.5 and incident heart failure differed across subgroups defined by diabetes, CKD, obesity, multimorbidity, or CRP level. Although point estimates appeared somewhat higher in some strata, confidence intervals overlapped and formal tests for interaction were consistently non-significant. These findings should be interpreted cautiously, as limited statistical power in some high-risk subgroups may have reduced our ability to detect heterogeneity. Therefore, our results do not support strong evidence of effect modification, but they also do not exclude the possibility of differential susceptibility in specific vulnerable populations. Several considerations may help explain this apparent lack of heterogeneity. First, the prevalence and severity of cardiometabolic disease in UK Biobank participants are lower than in the general population, which may have limited our ability to detect differential susceptibility. Second, vulnerability may be more continuous and multifactorial than can be captured by binary definitions such as diabetes or obesity. Third, PM2.5 may exert its effects through mechanisms that are not strongly dependent on baseline cardiometabolic status, such as autonomic or microvascular dysfunction.

Although the relative risks associated with PM2.5 were modest, the standardized absolute risk differences over 15 and 20 years were small and should be interpreted cautiously (25). This pattern suggests that the potential public health relevance of PM2.5 may be driven more by its widespread population exposure than by a large increase in absolute risk for any single individual. In addition, subgroup-specific absolute risk estimates were imprecise and should not be overinterpreted (26, 27). The major strengths of our study include its large sample size, long follow-up, detailed covariate information, and the ability to examine both relative and absolute risk measures. The use of standardized exposure assessment and linkage to national health records also enhances internal validity and completeness of outcome ascertainment. Although a small individual-level risk difference could still be relevant for a ubiquitous environmental exposure, our study did not directly estimate the population-attributable fraction or number of attributable heart failure cases; population-level implications should therefore be interpreted cautiously. The stronger PM2.5 estimate observed in the two-pollutant model should be interpreted cautiously. PM2.5 and NO2 were strongly correlated (Pearson r = 0.869), with variance inflation factors of 4.26 and 4.36, respectively, indicating appreciable but not extreme multicollinearity. In a two-pollutant model, the PM2.5 coefficient represents its association conditional on NO2 and may therefore differ materially from the single-pollutant estimate. The larger coefficient may reflect suppression or differences in shared spatial and source-related variation between the pollutants rather than a synergistic biological effect. Because we did not formally test a PM2.5 × NO2 interaction, these findings should not be interpreted as evidence of pollutant synergy.

Several limitations should be acknowledged. First, air pollution exposure was based on the 2010 annual average concentration assigned to participants’ residential addresses and was used as a proxy for near-baseline long-term ambient exposure. This approach did not account for residential relocation or temporal variation in ambient pollution during follow-up and may therefore have introduced exposure misclassification. Moreover, residential ambient PM2.5 does not capture total personal exposure from indoor, occupational, or commuting-related sources. The exposure metric should therefore be interpreted as a proxy for residential ambient exposure rather than individual cumulative PM2.5 dose. Second, UK Biobank participants are generally healthier and more socioeconomically advantaged than the general population, which may limit generalizability. Third, residual confounding by unmeasured or imperfectly measured factors cannot be excluded. Fourth, the fully adjusted analyses were restricted to participants with complete covariate information, resulting in a substantial reduction in sample size. If covariate completeness was related to socioeconomic, behavioral, or health characteristics associated with both exposure and heart-failure risk, complete-case analysis may have introduced selection bias; the direction and magnitude of this potential bias cannot be determined with certainty. Finally, limited event numbers in some high-risk subgroups reduced statistical power for interaction and subgroup-specific absolute risk analyses.

## 5 Conclusion

Long-term PM2.5 exposure was associated with a modest increase in incident heart failure risk in UK Biobank participants, whereas evidence for an association with ischemic stroke and for effect modification by cardiometabolic vulnerability was limited. These findings support a possible role of long-term PM2.5 exposure in heart failure risk, although the observed effect size was small and should be interpreted cautiously.

## Data Availability

The data analyzed in this study is subject to the following licenses/restrictions: data are available from the UK Biobank upon approved application. Requests to access these datasets should be directed to https://www.ukbiobank.ac.uk/.
