# Supplementary material for: Long-term PM2.5 exposure and incident heart failure in the UK Biobank: relative and absolute risk estimates
Source: Front Public Health. 2026 Sep 14;14:1864868. doi: 10.3389/fpubh.2026.1864868 (PMC13617081; doi:10.3389/fpubh.2026.1864868)
Supplement: Supplementary file 1 [file Table_1.docx]

**Table S1. Definitions of exposures, outcomes, covariates, and data sources**

| **Category** | **Variable** | **Definition / Processing** | **Data source** |
| --- | --- | --- | --- |
| Exposure | PM2.5 | Annual average PM2.5 concentration in 2010 at residential address; analysed per IQR and by quartiles (Q1–Q4) | UK Biobank air pollution model |
| Exposure | NO2 | Annual average nitrogen dioxide concentration in 2010; analysed per IQR | UK Biobank air pollution model |
| Exposure | PM10 | Annual average PM10 concentration in 2010; used in multipollutant score | UK Biobank air pollution model |
| Exposure | Multi-pollutant score | Mean of z-standardised PM2.5, NO2 and PM10 | Derived |
| Outcome | Heart failure | First hospital or death record with ICD-10 I50 after baseline | UKB inpatient + death registry |
| Outcome | Ischaemic stroke | First hospital record with ICD-10 I63 after baseline | UKB inpatient |
| Outcome | Censoring | Earliest of death or 31 Dec 2024 | UKB death registry |
| Covariate | Age | Age at recruitment (years, continuous) | UKB baseline |
| Covariate | Sex | Male / female | UKB baseline |
| Covariate | BMI | Body mass index at baseline (kg/m²) | UKB baseline |
| Covariate | Obesity | BMI ≥ 30 kg/m² | Derived |
| Covariate | Smoking | Never / former / current | UKB questionnaire |
| Covariate | Alcohol | Frequency of intake | UKB questionnaire |
| Covariate | Physical activity | MET-equivalent score | UKB questionnaire |
| Covariate | Townsend index | Socioeconomic deprivation index | UKB baseline |
| Covariate | CRP | Serum C-reactive protein (mg/L), dichotomised at median | UKB biomarker |
| Covariate | Diabetes | ICD-10 E10/E11 before baseline | UKB inpatient |
| Covariate | CKD | eGFR < 60 ml/min/1.73m² (CKD-EPI 2021) | Derived from creatinine |
| Covariate | Multimorbidity | ≥2 of obesity, diabetes, CKD | Derived |
| Sensitivity | Early event exclusion | Excluded events within 2 years of baseline | Analysis |
| Sensitivity | Competing risk | Death treated as competing event | Analysis |
