# Supplementary material for: Long-term PM2.5 exposure and incident heart failure in the UK Biobank: relative and absolute risk estimates
Source: Front Public Health. 2026 Sep 14;14:1864868. doi: 10.3389/fpubh.2026.1864868 (PMC13617081; doi:10.3389/fpubh.2026.1864868)
Supplement: Supplementary file 2 [file Table_2.docx]

**Table S2. PM2.5 quartiles (Q4 vs Q1) and trend**

| Outcome | Exposure | N | Events | HR (Q4 vs Q1) | 95% CI | P | P-trend |
| --- | --- | --- | --- | --- | --- | --- | --- |
| HF | PM2.5 quartiles | 149 410 | 4 964 | 1.133 | 1.037–1.238 | 0.006 | 0.005 |
| Stroke | PM2.5 quartiles | 149 410 | 3 795 | 1.005 | 0.910–1.110 | 0.924 | 0.676 |
