# Supplementary material for: Long-term PM2.5 exposure and incident heart failure in the UK Biobank: relative and absolute risk estimates
Source: Front Public Health. 2026 Sep 14;14:1864868. doi: 10.3389/fpubh.2026.1864868 (PMC13617081; doi:10.3389/fpubh.2026.1864868)
Supplement: Supplementary file 3 [file Table_3.docx]

**Table S3. Sequentially adjusted models**

| Outcome | Model | Exposure | N | Events | HR | 95% CI | P |
| --- | --- | --- | --- | --- | --- | --- | --- |
| HF | Model 1 | PM2.5 (per IQR) | 206 829 | 7 541 | 1.183 | 1.153–1.213 | <0.001 |
| HF | Model 1 | NO2 (per IQR) | 206 829 | 7 541 | 1.167 | 1.135–1.200 | <0.001 |
| HF | Model 1 | Multi-score (per 1 SD) | 206 829 | 7 541 | 1.137 | 1.112–1.162 | <0.001 |
| HF | Model 2 | PM2.5 (per IQR) | 159 659 | 5 372 | 1.022 | 0.986–1.058 | 0.234 |
| HF | Model 2 | NO2 (per IQR) | 159 659 | 5 372 | 0.974 | 0.936–1.013 | 0.181 |
| HF | Model 2 | Multi-score (per 1 SD) | 159 659 | 5 372 | 1.003 | 0.974–1.033 | 0.863 |
| HF | Model 3 | PM2.5 (per IQR) | 149 410 | 4 964 | 1.045 | 1.005–1.086 | 0.027 |
| HF | Model 3 | NO2 (per IQR) | 149 410 | 4 964 | 0.998 | 0.956–1.042 | 0.937 |
| HF | Model 3 | Multi-score (per 1 SD) | 149 410 | 4 964 | 1.021 | 0.986–1.058 | 0.242 |
| Stroke | Model 1 | PM2.5 (per IQR) | 206 829 | 5 596 | 1.137 | 1.103–1.172 | <0.001 |
| Stroke | Model 1 | NO2 (per IQR) | 206 829 | 5 596 | 1.125 | 1.089–1.163 | <0.001 |
| Stroke | Model 1 | Multi-score (per 1 SD) | 206 829 | 5 596 | 1.096 | 1.069–1.125 | <0.001 |
