# Supplementary material for: Long-term PM2.5 exposure and incident heart failure in the UK Biobank: relative and absolute risk estimates
Source: Front Public Health. 2026 Sep 14;14:1864868. doi: 10.3389/fpubh.2026.1864868 (PMC13617081; doi:10.3389/fpubh.2026.1864868)
Supplement: Supplementary file 4 [file Table_4.docx]

**Table S4. Effect modification of PM2.5 (per IQR) on incident heart failure by vulnerability factors**

| Modifier | N | Events | HR | 95% CI | P value | P for interaction |
| --- | --- | --- | --- | --- | --- | --- |
| No CKD | 147,542 | 4,701 | 1.048 | 1.007–1.091 | 0.020 | 0.444 |
| CKD | 1,868 | 263 | 1.018 | 0.846–1.226 | 0.849 | 0.444 |
| CRP (low) | 78,496 | 1,786 | 1.061 | 0.993–1.133 | 0.082 | 0.855 |
| CRP (high) | 70,914 | 3,178 | 1.039 | 0.990–1.090 | 0.122 | 0.855 |
| No Diabetes | 146,753 | 4,631 | 1.054 | 1.012–1.097 | 0.011 | 0.650 |
| Diabetes | 2,657 | 333 | 0.932 | 0.800–1.085 | 0.362 | 0.650 |
| Multimorbidity (<2) | 147,390 | 4,639 | 1.057 | 1.015–1.100 | 0.007 | 0.144 |
| Multimorbidity (≥2) | 2,020 | 325 | 0.898 | 0.766–1.054 | 0.189 | 0.144 |
| Obesity (BMI <30) | 115,855 | 3,013 | 1.042 | 0.991–1.095 | 0.112 | 0.522 |
| Obesity (BMI ≥30) | 33,555 | 1,951 | 1.052 | 0.989–1.120 | 0.106 | 0.522 |

**Notes:** Hazard ratios (HRs) are per interquartile range (IQR) increase in PM2.5, estimated from multivariable Cox models adjusted for age, sex, socioeconomic status, smoking, alcohol intake, physical activity, BMI, diabetes, CKD, CRP, and traffic-related covariates. P for interaction was obtained from multiplicative interaction terms between PM2.5 and each modifier.
