# Supplementary material for: Long-term PM2.5 exposure and incident heart failure in the UK Biobank: relative and absolute risk estimates
Source: Front Public Health. 2026 Sep 14;14:1864868. doi: 10.3389/fpubh.2026.1864868 (PMC13617081; doi:10.3389/fpubh.2026.1864868)
Supplement: Supplementary file 5 [file Table_5.docx]

**Table S5. Standardized 15- and 20-year absolute risks and absolute risk differences (per 1000 persons)**

| Group | N | Events | Horizon (years) | Risk in Q1 (per 1000) | 95% CI | Risk in Q4 (per 1000) | 95% CI | ARD (per 1000) | 95% CI |
| --- | --- | --- | --- | --- | --- | --- | --- | --- | --- |
| Overall | 149,410 | 4,964 | 15 | 0.009 | (0.007–0.014) | 0.011 | (0.007–0.016) | 0.001 | (0.000–0.002) |
| Diabetes | 2,657 | 333 | 15 | 0.274 | (0.051–1.163) | 0.221 | (0.043–0.868) | −0.053 | (−0.299–0.053) |
| Overall | 149,410 | 4,964 | 20 | 0.014 | (0.009–0.020) | 0.016 | (0.011–0.022) | 0.002 | (0.000–0.004) |
| Diabetes | 2,657 | 333 | 20 | 0.373 | (0.092–1.524) | 0.301 | (0.080–1.226) | −0.072 | (−0.442–0.033) |
