# Supplementary material for: Long-term PM2.5 exposure and incident heart failure in the UK Biobank: relative and absolute risk estimates
Source: Front Public Health. 2026 Sep 14;14:1864868. doi: 10.3389/fpubh.2026.1864868 (PMC13617081; doi:10.3389/fpubh.2026.1864868)
Supplement: Supplementary file 6 [file Table_6.docx]

**Table S6. Sensitivity analyses (HF outcome, PM2.5 per IQR)**

| Analysis | N | Events | HR | 95% CI | P |
| --- | --- | --- | --- | --- | --- |
| Exclude events within 2 years | 149 213 | 4 767 | 1.035 | 0.994–1.077 | 0.092 |
| Never smokers only | 83 038 | 2 045 | 1.054 | 0.992–1.121 | 0.091 |
| Two-pollutant model (PM2.5 + NO2) | 149 410 | 4 964 | 1.142 | 1.068–1.221 | <0.001 |
| Restrict diabetes = 0 | 146 753 | 4 631 | 1.054 | 1.012–1.097 | 0.011 |
| Restrict CKD = 0 | 147 542 | 4 701 | 1.048 | 1.007–1.091 | 0.020 |
